# ^18^F‑FDG PET/CT based radiomics features improve prediction of prognosis: multiple machine learning algorithms and multimodality applications for multiple myeloma

**DOI:** 10.1186/s12880-023-01033-2

**Published:** 2023-06-27

**Authors:** Haoshu Zhong, Delong Huang, Junhao Wu, Xiaomin Chen, Yue Chen, Chunlan Huang

**Affiliations:** 1 Department of Hematology, the Affiliated Hospital of Southwest Medical University, Luzhou City, Sichuan China; 2 Stem Cell Laboratory, The Clinical Research Institute, Affiliated Hospital of Southwest Medical University, Luzhou City, Sichuan China; 3 Southwest Medical University, Luzhou City, Sichuan China; 4 Department of Nuclear Medicine & PET Center, Huashan Hospital, Fudan University, Shanghai, 200040 China; 5 Department of Nuclear Medicine, the Affiliated Hospital of Southwest Medical University, Luzhou City, Sichuan China

**Keywords:** Multiple myeloma, Prognostic prediction, ^18^F‑FDG PET/CT, Machine-learning, Radiomics

## Abstract

**Purpose:**

Multiple myeloma (MM), the second most hematological malignancy, have been studied extensively in the prognosis of the clinical parameters, however there are only a few studies have discussed the role of dual modalities and multiple algorithms of ^18^F-FDG (^18^F-fluorodeoxyglucose) PET/CT based radiomics signatures for prognosis in MM patients. We hope to deeply mine the utility of raiomics data in the prognosis of MM.

**Methods:**

We extensively explored the predictive ability and clinical decision-making ability of different combination image data of PET, CT, clinical parameters and six machine learning algorithms, Cox proportional hazards model (Cox), linear gradient boosting models based on Cox’s partial likelihood (GB-Cox), Cox model by likelihood based boosting (CoxBoost), generalized boosted regression modelling (GBM), random forests for survival model (RFS) and support vector regression for censored data model (SVCR). And the model evaluation methods include Harrell concordance index, time dependent receiver operating characteristic (ROC) curve, and decision curve analysis (DCA).

**Results:**

We finally confirmed 5 PET based features, and 4 CT based features, as well as 6 clinical derived features significantly related to progression free survival (PFS) and we included them in the model construction. In various modalities combinations, RSF and GBM algorithms significantly improved the accuracy and clinical net benefit of predicting prognosis compared with other algorithms. For all combinations of various modalities based models, single-modality PET based prognostic models’ performance was outperformed baseline clinical parameters based models, while the performance of models of PET and CT combined with clinical parameters was significantly improved in various algorithms.

**Conclusion:**

^18^F‑FDG PET/CT based radiomics models implemented with machine learning algorithms can significantly improve the clinical prediction of progress and increased clinical benefits providing prospects for clinical prognostic stratification for precision treatment as well as new research areas.

**Supplementary Information:**

The online version contains supplementary material available at 10.1186/s12880-023-01033-2.

## Background

The second most prevalent hematologic malignancy is MM. It is a plasma cell disorder characterized by aberrant monoclonal plasma cell (PC) proliferation in bone marrow (BM) [1]. It develops from monoclonal gammopathy of undetermined significance (MGUS), and undergoes an intermediate phase known as smoldering MM (SMM), before advancing to active MM [2].

At present, MM is diagnosed using laboratory- and image-based assessments. The most widely utilized imaging methods for MM diagnosis are CT and PET/CT. The bone and extraosseous symptoms of MM can be evaluated using PET/CT in terms of their presence, size, and metabolic activity [3]. 20% of newly diagnosed MM patients still have a dismal prognosis, despite recent improvements in new therapies for the survival of MM patients [4, 5]. Therefore, predicting MM patient prognosis and treating them with precision can potentially enhance patients' survival in the future [6]. In the clinics, cytogenetic examinations using bone marrow biopsy and aspiration are necessary for the prognostic stratification and precise treatment of MM patients [7]. However, this invasive procedure is often painful for the patients, and samples are difficult to obtain. There are times when an intrusive biopsy is ineffective on the first try and requires subsequent biopsies. Additionally, due to the heterogeneity of the obtained biopsy material, it may not be typical of the whole malignancy parts and only represents a tiny fraction of it.

Due to the limitations of conventional approaches outlined above, finding noninvasive ways of prognostic prediction among MM patients is a topic of significant research interest. There is a recent rise in using machine learning (ML) algorithms to predict patient prognosis based on radiomics features. This is a noninvasive and accurate mean of prognostic stratification, and it was previously reported in MM patients [8]. Schenone et al. employed ML algorithms to accurately stratify the outcome of autologous transplanted MM patients based on CT radiomics features. This, in turn, assisted in designing proper personalized treatments for individual patients [9]. Although the Li et al. study analyzed an ML model constructed from MRI-based radiomics features, in combination with a clinical model for MM, MRI is typically not the examination of choice for MM patients, and certain shortcomings still exist in this regard [3, 10]. Given these evidences, it is imperative to identify novel biomarkers for the accurate prediction of MM patient prognosis. Based on prior research, radiomics-based prognostic signatures have great potential in stratifying MM patients as either low- or high-risk. This information is crucial, particularly, for high-risk patients, who, with advanced therapy, may experience enhanced outcomes.

Furthermore, a multimodal application of ML has emerged in recent years that integrates radiomics features from PET, CT, and so on to construct ML models. This approach facilitates a closer examination of the entire intra-heterogeneous tumor, rather than the limited information gathered from unimodal medical images [11]. Haider et al. used 5 machine learning algorithms and multimodal model integrating both PET and CT features to analyze the relationship between oropharyngeal squamous cell carcinoma and human papillomavirus, with an area under the AUC of 0.78 [12]. Although there are related studies that predicted MM patient prognosis based on PET/CT imaging radiomics features. However, to date, there are no reports on employing the multimodal radiomics features of PET and CT and multiple ML algorithms to predict MM patient prognosis. Hence, herein, we utilized the aforementioned method to study prognosis and to enhance personalized patient care in order to better MM patient outcome [13, 14].

## Materials and methods

### Study population and clinical data

Patients with histopathologically proven MM who received a ^18^F-FDG PET/CT scan at our institution between February 2014 and October 2022 were involved in this retrospective analysis. Patients were included if: 1. Bone marrow samples that met the diagnostic criteria of the IMWG for multiple myeloma [15] were positive for the disease. 2. All patients' complete clinical data were accessible. 3. Pre-treatment ^18^F-FDG PET/CT was performed. Patients were disqualified if: 1. They have already had chemotherapy or autologous stem cell treatment before image scanning. 2. Image that clearly have artifacts. 3. Patients who also have other cancerous conditions. 4. The liver's mean standardized uptake value (SUVmean) is outside the range of 1.3 to 3.0 [16]. Clinical variables including gender, age at diagnosis, serum LDH level, Hemoglobin level, Globulin level, Albumin level, A/G level, Creatinine level, Glomerular filtration rate (GFR) level, calcium level, HCT level, Absolute Neutrophil count, Leukocytes count, red blood cells count, serum β2-microglobulin (B2M) level, R-ISS stage, ISS stage, cytogenetic abnormalities, and the patients' individual regimens arms were observed. The term "progression-free survival" refers to the time between the original diagnosis and any subsequent progression, relapse, or death. And the criterias of progression and relapse are based on the International Myeloma Working Group consensus Criteria for Response and Minimal Residual Disease Assessment in Multiple Myeloma [17].

### Image acquisition

From participating sites, we collected baseline ^18^F-FDG PET/CT images in the DICOM format. Patients with normal blood glucose levels were asked to fast, stop receiving intravenous glucose, and avoid severe activity or extended exercise for 6 h before intravenous ^18^F-FDG (3.7 MBq/kg) delivery. A hybrid PET/CT scanner (uMI780, United Imaging Healthcare, Shanghai, China) was used for all PET/CT imaging which included a low-dose CT scan (current 120 mA; tube voltage 120 kV; matrix 512 × 512 pixels; slice thickness 3.00 mm; window width 300–500 HU; window level 40–60 HU) and a PET scan (with 1.5 min/position in 3D acquisition mode and 5–6 bed positions), and it performed less than one hour after radiotracer injection as part of the scanning procedure. The PET image was reconstructed using attenuation iterative correction approach (Ordered Subsets Expectation Maximization, OSEM) and the window width and window level of the CT images were set to 350 and 50 then submitted to the MedEx workstation together with fusion imaging once all the patient's PET/CT image parameters had been standardized.

### Image segmentation and feature extraction

**Fig. 1:**
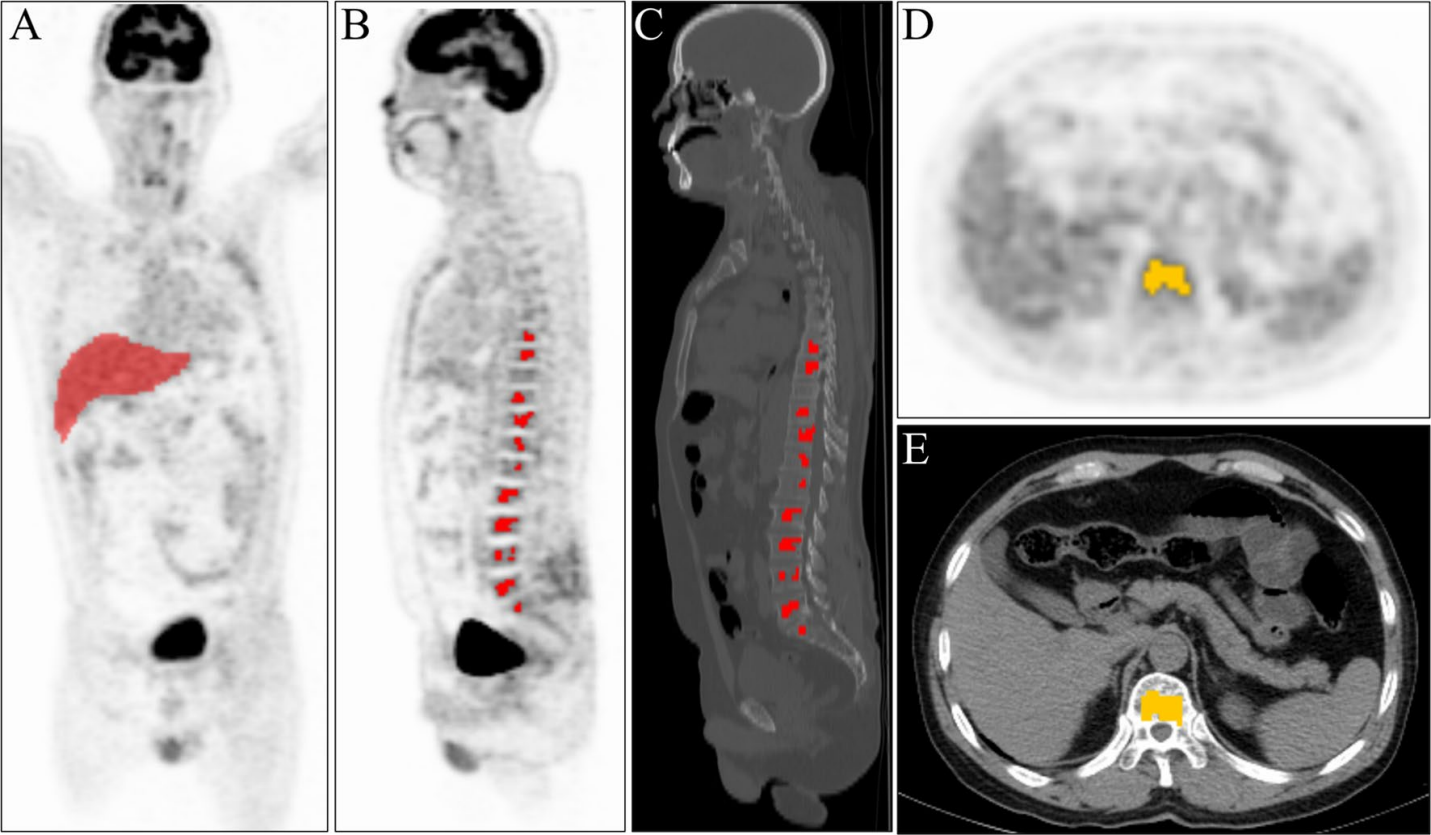
An example of delineation of VOIs and the outline of the gross liver of a 73-years-old male diagnosed with MM and ISS staging II, RISS staging II

The radiomic profiles were obtained from the semi-automatic delineation volumes of interest (VOIs) following normalization, discretion, and image resampling. To minimize the impact of varying slice thicknesses and voxel sizes on the radiomics profile, decrease dependence of the radiomic profiles on voxel size, and ensure rotational invariance of textural profiles, while retaining the original intensity scale and meaning of voxel values, two distinct discretization strategies were employed for the two quantitative functional imaging modalities (^18^F-FDG -PET and low dose CT) with fixed bin width (FBW) sizes (bin sizes for PET = 0.25; CT = 25) which may be more suitable in certain circumstances than a fixed bin number for comparative analysis by various modalities [18]. Based on the Image Biomarker Standardization Initiative (IBSI) criteria of image normalization and contrast prioritization inside VOIs, we employed tri-linear interpolation to produce isotropic 3 × 3 × 3 and 2 × 2 × 2 mm voxels in PET and CT scans respectively. An example of delineations of VOIs and the outline of the liver is shown in Fig. 1.

The radiomics features included Shape, the First Order Statistics (firstorder), Gray Level Cooccurence Matrix (glcm), Gray Level Dependence Matrix (gldm), Gray Level Run Length Matrix (glrlm), Gray Level Size Zone Matrix (glszm), and Neighbouring Gray Tone Difference Matrix (ngtdm). The image type includes, original image, wavelet filtering, square filtering, square root filtering, logarithm filtering, exponential filtering, gradient filtering transformed images, and LocalBinaryPattern2D, LocalBinaryPattern3D image types. These algorithms for obtaining radiomics features were according to the IBSI criteria [19].

PET and CT imaging were assessed and delineated by a nuclear medicine physician and verified by another nuclear radiologist with 13 years of extensive experience who was blind to the patients' prognosis. The LIFEx(version 7.3.0;https://www.lifexsoft.org/) [20] software was used to semi-automatically outline the VOIs which implemented a semi-automatic MTV protocol using a fixed threshold of 41% SUVmax and minimum absolute SUV 2.5 [16]. Bone marrow was considered involved if focal or multifocal lesions presented higher SUV than 1.5 (ratio of voxel SUV/liver SUVmax) uptake than the liver [21]. And gross liver was been manually delineated before the VOIs has been semiautomatically delineated. Non-tumor areas were manually erased (e.g., kidney, bladder, brain tissue, trachea, dental cavities, arthritis).

For each patient, SUVmax, MTV, TLG, sMTV, sTLG were recorded. The VOIs were then applied to CT images by overlaid onto co-registered CT scans and non-bone areas in CT images such as muscles, air, and surrounding fat planes were manually erased. A total of 1688 radiomic features from were extracted via PyRadiomics software(version 3.0.1) [22], and the Z-transform approach was used to normalize the obtained radiomics feature values in order to eliminate the magnitude disparity.

### Feature selection

Both CT, PET and clinical data were used feature screening special strategies as followed:1. To make the process of feature selection more clinically interpretable, we used univariable cox regression for variable screening with a threshold *p*-value < 0.05. 2. LASSO-penalized COX regression were used to process high-dimensional radiomics data of CT and PET by compressing the coefficient with different penalty parameter λ, a fivefold cross validation was performed to determine the optimal value of Lasso penalty parameter λ. 3. Subsequently stepwise model selection with using the Alkaike information criterion (AIC) defined final key features in clinical data, PET, CT data respectively, meanwhile verified the effects of multicollinearity of features selected using threshold Variance Inflation Factor (VIF) < 5. The VIF measures the severity of multicollinearity in multiple regression models [23]. It represents the ratio of the estimator variance of the regression coefficient to the variance when no linear correlation between the independent variables is assumed.

### Model construction

We further investigated the impact of 6 ML methods, namely, Cox, GB-Cox [24], CoxBoost [25], GBM [26], RFS [27], and SVCR [28, 29]. The R software 4.2.0 was employed for all ML method implementations. The particular details of R packages involved in this research ML methods are summarized in the Additional file: Supplementary Table 4.

### Tunning of hyperparameters

The adjustment of parameters for sophisticated models like the GBM and RFS took a lot of effort, while the Cox approach did not require parameterization, so the hyperparameters that affects the effect of the model the most is selected. And some of these algorithms have similar parameters such as number of trees and number of boosting steps so they are also in the scope of our tuning hyperparameters, and the range of hyperparameters are available at Supplementary Table 4. For each ML technique, the parameters were chosen from the combination of parameters that gave the greatest performance using the fivefold cross validation on a training fold with a grid search method.

### Evaluation of multiple machine learning algorithms and multimodality models

To evaluate the effectiveness of various ML approaches on the resampled training/validation groups, the Harrell concordance index (C-index) analysis with confidence interval (CI) was utilized. The percentage of CI was 95% in this study. We used timeROC package to draw time dependent receiver operating characteristic (ROC) curve to study the accuracy of various models to predict from 100 to 1600 days progress free time [30]. The clinical application prospects were analyzed with DCA nomogram, decision curve analysis (DCA) evaluated the clinical utility of different decision strategies was performed by calculating the net benefits for a range of threshold probabilities in the median follow-up time [31].

### Statistical analysis

Due of the small dataset size, we created training and validation groups using the bootstrap resampling approach (iterative resampling with replacement for 1000 times). The C-index obtained from resampled training and validation sets after 1000-times bootstraps. Time dependent ROC curves and DCA nomograms were obtained from the merged validation groups for each machine learning methods and modality combinations. The models were constructed on the training groups and internally validated using 1000 bootstrap samples to avoid overfitting. Besides, the survival curves evaluated the discontinuous variables by the Kaplan–Meier algorithm and significance was compared by the log-rank tests between groups. Spearman’s rank-order correlation was used to measure correlation-ship between two continuous variables which do not obey the normal distribution, while Pearson’s correlation was used for normal distribution continuous variables. Comparisons of C-index were performed for each 1000 resampling and on the fused 1000 resampling validation set data by Student’s t-test and a one-shot nonparametric approach respectively. Continuous variables are described using means and standard deviations, and categorical variables are demonstrated with percentages.

## Results

### Study samples

The 121 patients with MM who underwent ^18^F-FDG PET/CT exams at our facility between February 2014 and October 2022 were initially included in this retrospective study. However, 98 patients were ultimately enrolled in this study after meeting the inclusion and exclusion criteria. The median PFS time were 25.9 months (95% CI, 23.8—29.5 months). And the baseline clinical characteristics of the included patients were summarized in Table 1.

**Table 1:** Baseline clinical characteristics of patients

Characteristics	Level	Statistic
Gender	female	48 (49.0%)
	male	50 (51.0%)
Age_at_diagnosis	years	60.0 (± 10.6)
LDH	U/L	222.1 (± 128.5)
Hemoglobin	g/L	95.2 (± 24.3)
Globulins	g/L	50.0 (± 27.8)
Albumin	g/L	37.0 (± 7.4)
A/G ratio		1.1 (± 0.7)
Creatinine	μmol/L	115.6 (± 109.3)
GFR	ml/min	77.5 (± 51.5)
Calcium	mmol/L	2.5 (± 0.4)
HCT		0.3 (± 0.1)
Absolute Neutrophils	10^9/L	3.5 (± 1.7)
Leukocytes	10^9/L	5.7 (± 2.2)
Red blood cells	10^9/L	3.2 (± 0.8)
B2M	mg/L	7.3 (± 6.2)
ISS_staging	I	25 (25.5%)
	II	29 (29.6%)
	III	44 (44.9%)
Adverse prognostic cytogenetics status	No	57 (58.2%)
	yes	41 (41.8%)
RISS_staging	I	23 (23.5%)
	II	46 (46.9%)
	III	29 (29.6%)

*Riss* Revised-International Staging System based staging, *B2M* β2-microglobulin, *ISS* International Staging System, *HCT* Hematocrit, *A/G ratio* Albumin/ Globulin (Ratio), *GFR* Glomerular filtration rate, *LDH* Lactate dehydrogenase

Continuous variables are represented by the mean ± standard deviation, while categorical variables are expressed as percentages

Adverse prognosis cytogenetics Status: FISH analysis confirmed del(17p), t (4;14), t (14;16), add 1q21;

### Feature selection

**Fig. 2:**
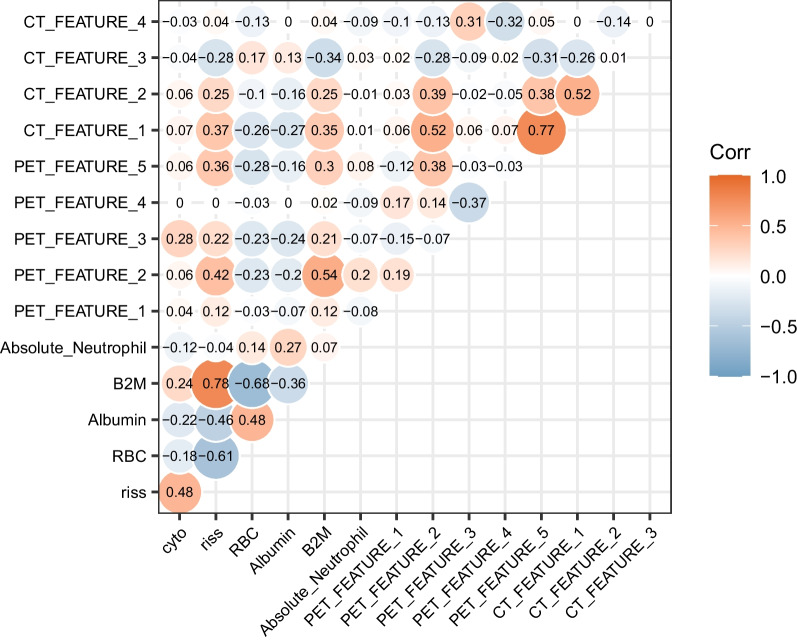
A heatmap illustrates correlation coefficient among the finalized features from PET, CT, and clinical parameters. #PET_FEATURE_1: wavelet.LLL_firstorder_10Percentile. #PET_FEATURE_2: exponential_glcm_InverseVariance. #PET_FEATURE_3: lbp.2D_firstorder_InterquartileRange. #PET_FEATURE_4: lbp.3D.k_glcm_MCC. #PET_FEATURE_5: original_shape_MajorAxisLength. #CT_FEATURE_1: original_shape_MinorAxisLength. #CT_FEATURE_2: original_ngtdm_Busyness. #CT_FEATURE_3: exponential_glszm_SmallAreaLowGrayLevelEmphasis. #CT_FEATURE_4: wavelet.HLH_gldm_SmallDependenceEmphasis

Firstly, by univariate Cox proportional regression analyses, and the threshold for *p*-value was less than 0.05, we screened 11 clinical parameters, 266 features derived from PET radiomics features, and 408 features derived from CT which were significantly associated with PFS. Then lasso-cox regression with optical lasso with penalty parameters λ screened 10 PET-based radiomics features, 7 CT-based radiomics features (Supplementary Table 1/ Supplementary Fig. 1). Finally, we used the AIC to select finalized features via a step-down backward process from the multivariate regression model, and 5 PET-based features, and 4 CT based features, as well as 6 clinically derived features, were finally identified (Table 2). The final VIF values and the correlation heatmap verified that there was no significant covariance among the final 15 identified features (Fig. 2/Table 2). In addition, features including ISS_staging, RISS_staging, and adverse prognostic cytogenetics status which were from clinical parameters were evaluated by Kaplan–Meier analyses. (Supplementary Fig. 2).

**Table 2:** Finalized features screened from the step 3 incorporated in the model construction

Feature_names	VIF	HR	*P*-value
Adverse prognostic cytogenetics status	2.633	2.197	0.00018
RISS_Staging	3.645	2.482	0.00009
Red blood cells	4.372	0.613	0.01988
Albumin	4.173	0.447	0.00009
B2M	3.687	1.426	0.00472
Absolute Neutrophils	2.336	0.597	0.03329
wavelet.LLL_firstorder_10Percentile_PET	2.48	1.796	0.00396
exponential_glcm_InverseVariance_PET	2.766	1.995	0.00068
lbp.2D_firstorder_InterquartileRange_PET	4.352	1.470	0.00569
lbp.3D.k_glcm_MCC_PET	2.307	1.479	0.03082
original_shape_MajorAxisLength_PET	4.638	1.777	0.00408
original_shape_MinorAxisLength_CT	3.794	1.704	0.00233
original_ngtdm_Busyness_CT	3.335	1.378	0.00529
exponential_glszm_SmallAreaLowGrayLevelEmphasis_CT	1.52	0.492	0.00273
wavelet.HLH_gldm_SmallDependenceEmphasis_CT	1.765	0.552	0.01432

*Riss* Revised-International Staging System based staging, *B2M* β2-microglobulin, *ISS* International Staging System, *HCT* Hematocrit, *A/G ratio* Albumin/ Globulin (Ratio), *GFR* Glomerular filtration rate, *LDH* Lactate dehydrogenase

Adverse prognosis cytogenetics Status: FISH analysis confirmed del(17p), t (4;14), t (14;16), add 1q21

**Fig. 3:**
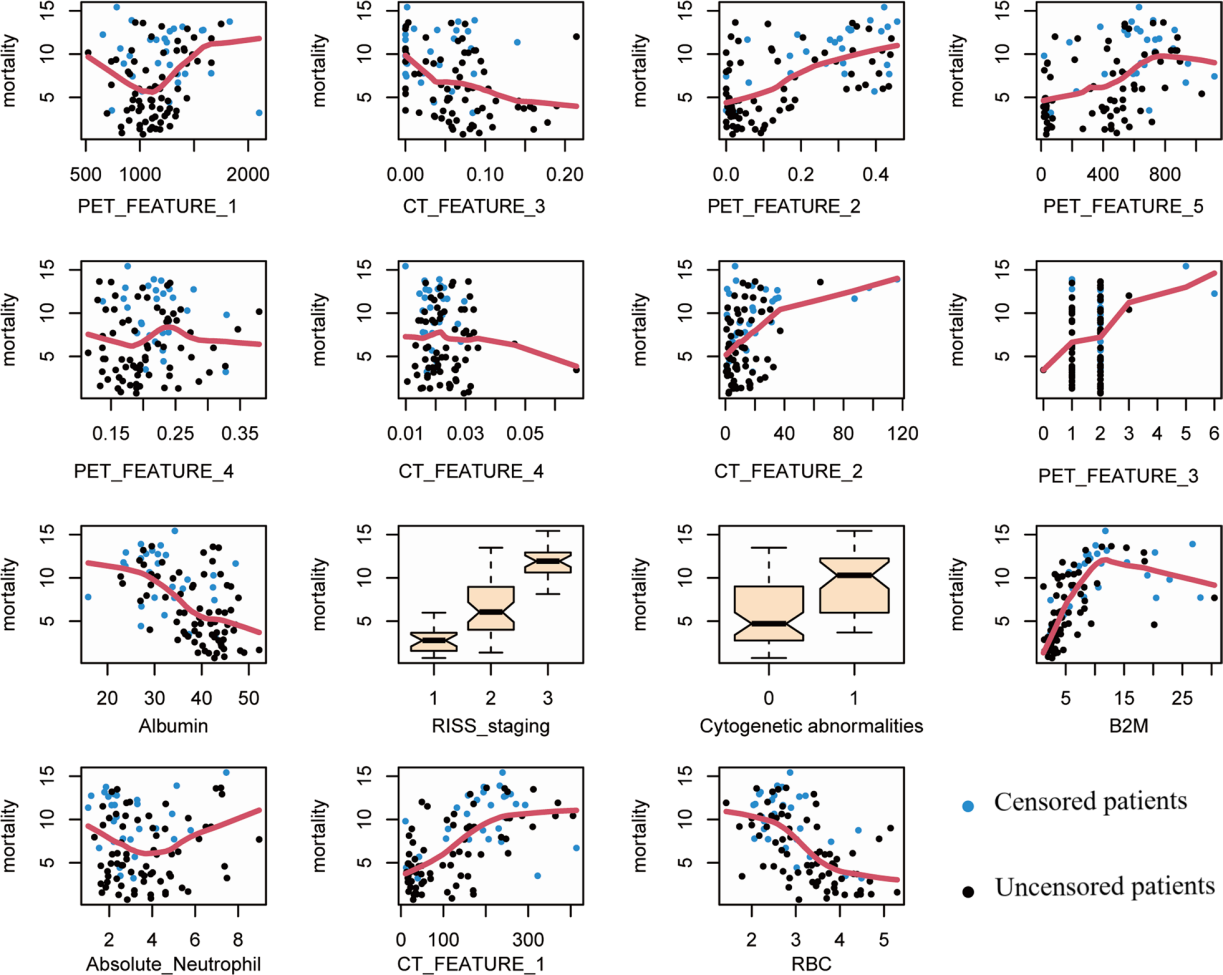
Random forests for survival model based estimations of relationship of finalized features’ level with mortality (PFS outcomes). The vertical axis displays the ensemble predicted value (mortality representing estimated risk for an individual), while x-variables (clinical, PET and CT based features) are plotted on the horizontal axis

To make the screened features clinically interpretable, we used two methods to assess the impact of features on patient prognosis. 1. Hazard ratio (HR) based on a linear equation of the Cox proportional hazards model. HR denotes the odds ratio of individual features in the linear equation, reflecting the speed of the occurrences of outcome events, greater than 1 indicates the faster the time to the occurrence of the outcomes (Table 2). 2. Nonlinear model based on random forests for a survival model, it supplies a detailed insight into the nonlinear relationship of each characteristic' level concerning mortality (PFS outcomes), and the mortality was calculated on the median duration of follow-up (Fig. 3).

### Assessment and comparison of models

The training and validation sets are generated by 1000 iterations of resamples implemented with the “bootstrap” method, and on the training and validation sets, the model's C-index is evaluated. We identified whether there were statistical differences for CT, CT + CLI, PET, and PET + CLI modalities when compared with clinical parameters respectively in the fused 1000 validating groups. And both statistical methods (Student’s t-test and a one-shot nonparametric test) showed significance (*p* < 0.001) in all 6 algorithms except for the SVRC algorithm constructed PET based radiomics model when compared with clinical parameters based model (*P =* 0.4824: a one-shot non-parametric test, *P =* 0.3468: Student’s t-test).

**Fig. 4:**
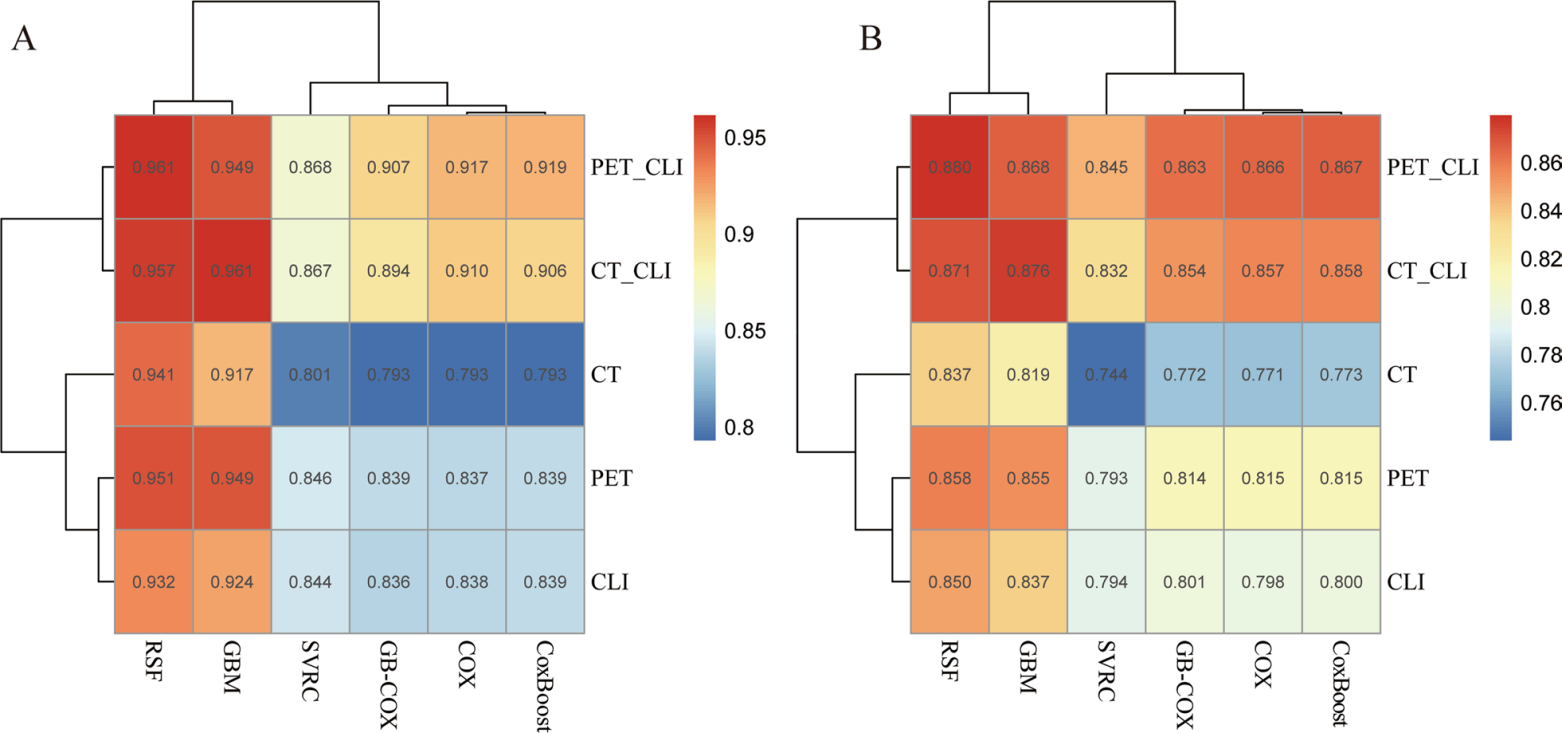
**A**-**B** Heatmaps with clustering illustrates the average performance of predictive ability of different algorithms and different modalities of 1000 bootstrap iterations in training groups and validation groups respectively.The similar performance of modalities and algorithms are considered to belong to the same cluster in the clustering analysis

The pictures depicted the performance of 6 ML methods (in columns) and 5 different modalities combinations (in rows) on the 1000 times merged training/validation bootstrap sampling (Fig. 4). It's interesting to observe that the best model was the combined model of the RSF algorithm, clinical parameters, and PET-based radiomics features in validation groups (Average CI: 0.880, 95% CI: 0.878–0.881) (Supplementary Tables 2 and 3), and the model of the combination of GBM algorithm, clinical parameters, and CT based radiomics had a leading C-index in training groups respectively. (Average CI: 0.961, 95% CI: 0.961–0.962) (Supplementary Tables 2 and 3).

And a heatmap with clustering showed the average C-index of various combinations, indicating that GBM and RSF algorithms performed consistently, and Cox and CoxBoost algorithms performed comparably but not as well as the former two algorithms. The combination of clinical parameters with PET or CT is more accurate than either one alone to predict the PFS for myeloma (Fig. 4).

**Fig. 5:**
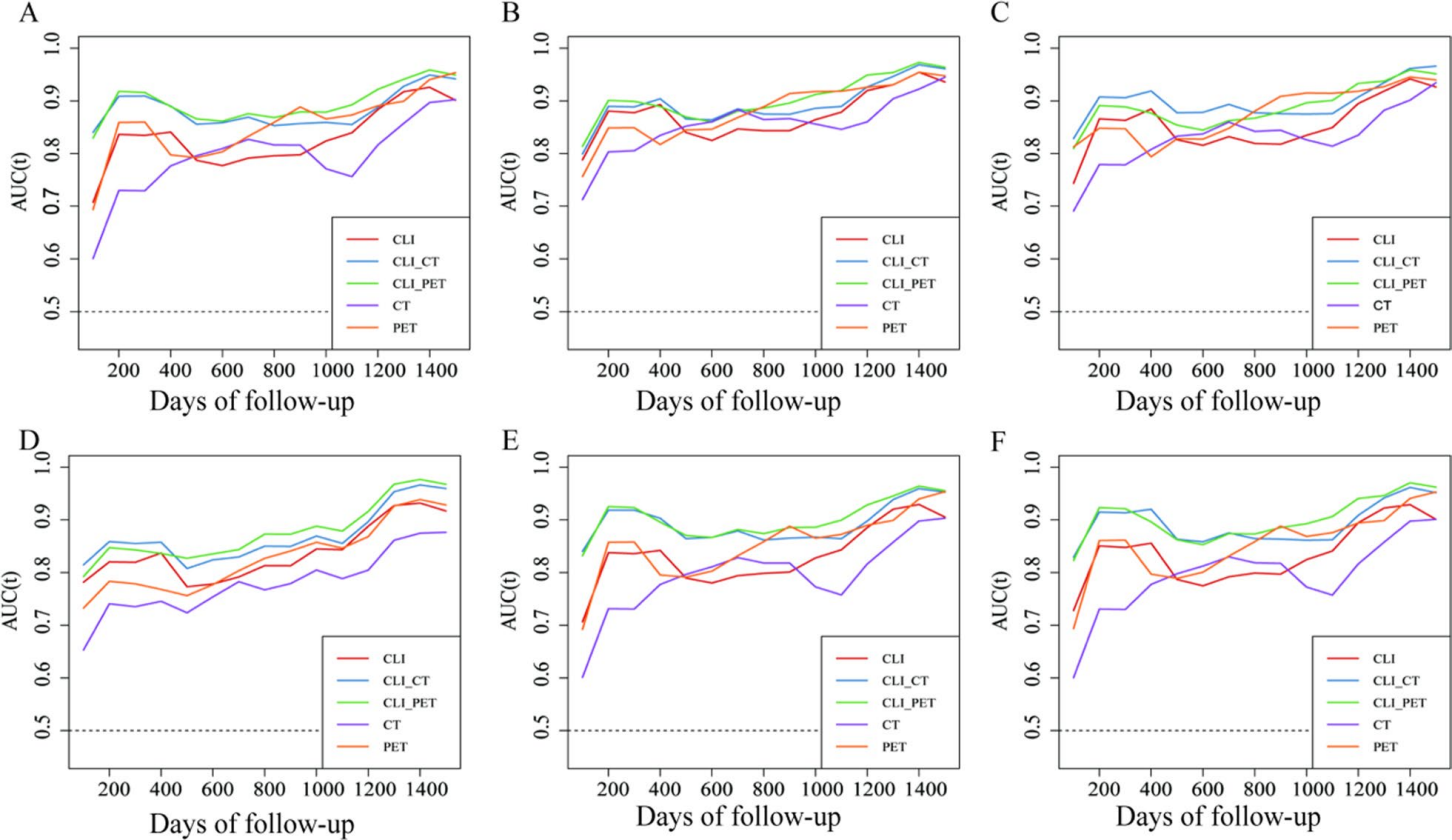
**A**-**F** time dependent ROC nomogram depicted the performance for predicting PFS of Cox, RSF, GBM, SVRC, CoxBoost, GB-COX algorithms respectively. The vertical axis displays the area under the receiver operating characteristic curve (AUC), and time of follow-up are plotted on the horizontal axis

The time ROC depicts the change in AUC values within the varying in follow-up time (Fig. 5). Time-dependent ROC analysis indicated that for each algorithm, performance for the prediction of prognosis can be greatly improved with the addition of PET or CT features to clinical parameters during the follow-up time. Single-modality PET models demonstrated higher prediction accuracy than clinical parameters in the late during the mid- to late-term follow-up time.

**Fig. 6:**
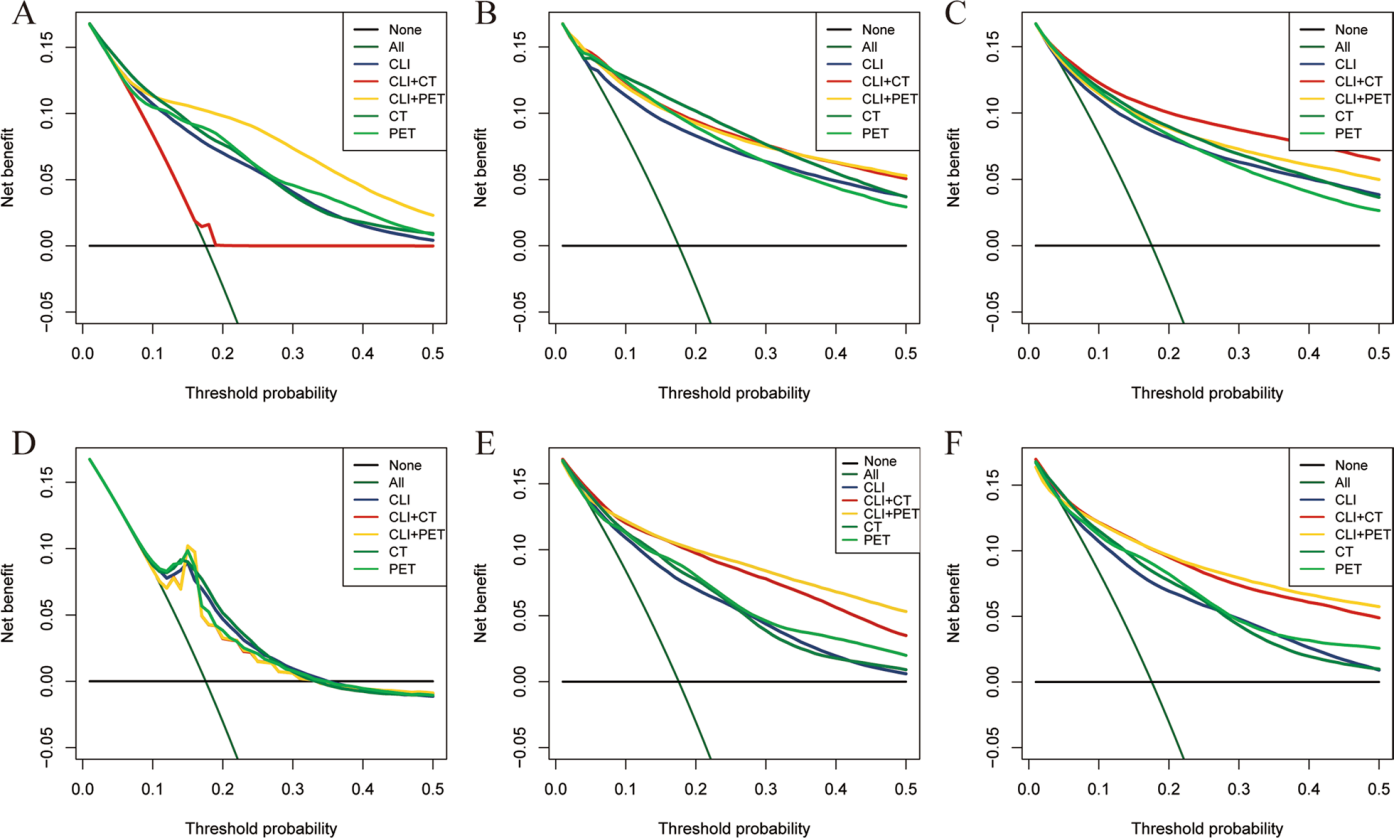
**A**-**F** DCA nomogram depicted the clinical net benefit of treat all, none treatment, and treatment of different modalities for Cox, RSF, GBM, SVRC, CoxBoost, GB-COX algorithms respectively

The decision curve analysis for the individualized prediction models is presented in Fig. 6, decision curve showed that except for the DCA curve of the SVRC algorithm and the combination CLI + CT in COX regression, both CT + PET and PET + CLI improve the clinical decision-making for patients, the threshold probability based on CT + PET and PET + CLI was better than that in the clinical strategies. However, The DCA of CT- or PET-based models alone does not exhibit a higher net benefit of decision-based on nomogram compared to clinical parameters based models.

## Discussion

At present, the role of ^18^F-FDG PET/CT in diagnostics and response evaluation criteria among MM patients has reached an extremely significant level of evidence for clinical decision making, prognosis determination, and treatment response evaluation [32–34]. However, only a few prior studies have examined the prognostic values of radiomics features in MM. Using the Cox regression model, Yang et al. confirmed that the radiomics profiles of bone marrow MRI exhibit obvious correlation with MM patient OS. Moreover, the predictive performance of radiomics-based signature is far superior to the traditional clinical model [10]. Ludivine Morvan et al. provided a novel radiomics feature selection protocol for ^18^F-FDG PET-based radiomics in MM patients, and they emphasized the advantages of employing image-based characteristics (including, textural profiles) for disease progression estimation [35]. In addition to the prognostic investigations, ^18^F-FDG PET/CT-based radiomics have been used in other different application of areas in MM. In comparison to human specialists, the radiomics model showed a significant improvement in diagnostic capacity. For example, the PET radiomics measure is quite effective in differentiating between bone metastases and vertebral MM [36]. However, to our knowledge, this report is the first to utilize CT and PET-based radiomics features, as well as combined clinical parameters implemented with multiple ML methods, to predict prognosis of MM patients. We employed a total of 10 image types and 8 features types in this study, and fully explored the radiomics features of PET/CT in MM patients in detail.

The bootstrap method was used in this study. Bootstrap is a common statistical method that uses repeated resampling of data samples to generate larger sample sets, thereby avoiding the limitation of small sample sizes. Similar bootstrap procedures were implemented in studies of Yilong Huang et al. [37], Wen L et al. [38], and Mostafa Nazari et al. [39]The significant differences between the clinical models and the radiomics models were compared using two statistical methods [40]. Using multiple feature screening methods, we retained the features with clinical interpretability while eliminating the multicollinearity among features.

Based on our analysis, the late RISS, ISS staging, high-risk genetic status, anemia, high serum globulin level, and low albumin levels were strongly associated with shorter PFS, which corroborates with published clinical trials and findings within clinical practice [34, 41]. PET-based features original_shape_MinorAxisLength, original_shape_LeastAxisLength_PET, MTV, sMTV demonstrated relevancy of PFS in the step1 for MM progress in this study. However, they were excluded from analysis during the feature selection step 2 due to the multicollinearity because the coefficients became 0 at the optimal penalty parameter. Our rationale was that these features simultaneously reflected high tumor burden and late tumor stage, and thus, depicted the magnitudes of the tumors.

The exclusion of CT-based features such as wavelet.HHH_gldm_SmallDependenceEmphasis and wavelet.HLH_gldm_SmallDependenceEmphasisCT was due to their extraction process from the same feature type of radiomics feature and the same type of filter of image, with only different combinations generated by using high-pass and low-pass filters in each of the three dimensions. Thus, these features also exhibit high collinearity and were therefore excluded from the analysis.

Other factors that are known to impact MM prognosis are high level LDH, hypercalcemia, renal insufficiency, and baseline treatment arms, TLG and sTLG. However, in this study, these factors did not reach significance likely due to the limited data and unintentional selection bias. It is also possible that these factors have less prognostic significance, compared to the other factors identified in this report, which is similar to the findings of the Yang Li et al. and Bastien Jamet et al. studies [10–13].

Using RSF and GBM algorithms, we further enhanced PFS accuracy in various modalities, and improved the overall clinical decision-making process. However, the overall GB-COX and CoxBoost algorithms accuracies did not improve significantly, compared to the multivariate COX regression models, yet they significantly improved the clinical decision-making ability in the combinations of PET + CLI and CT + CLI. Unlike the boosting-based algorithm,the decision tree-based algorithm RSF/GBM was more prone to overfitting producing higher differences between the bootstrap training sets and the validation sets. However, the SVRC algorithm achieved the lowest predictive performance in our systematic evaluation likely due to our feature selection steps, as such, this requires further exploration in future investigations [35, 42, 43]. Furthermore, in this investigation, we observed that the PET-based radiomics features were better in predicting MM patient prognosis, compared to CT-based radiomics features. We also demonstrated that integrating the radiomics signature with patient clinical profile greatly enhanced the prognostic predictive ability of our model. Based on this evidence, the radiomics signature is critical for estimating patient prognosis. It not only provides prognostic efficiency of bone marrow PET/CT radiomics in MM patients, but also has potential for clinical risk classification. This report offers an essential supplementary reference for radiomics-based prognostic models as we compared numerous ML methods for PFS estimation of MM patients. This large-scale comparison is beneficial for the accurate selection of ML methods for radiomics-based PFS estimation.

There were several restrictions on this work. First of all, because it was a single-center retrospective study, there could have been accidental bias in the patient selection process. As a result, our conclusions are not representative of the general population. Secondly, owing to a relatively small patient population and absence of an external groups for validation, we employed the bootstrap technique for model assessment to circumvent the issue of limited sample population. We recommend that future investigations assess the proposed prognostic model among a large multicenter-recruited patient population, and validate the results in an external independent validation cohort.

## Conclusion

^18^F-FDG PET/CT based radiomics models implemented with machine learning algorithms can significantly improve the clinical prediction of progress and increased clinical benefits providing prospects for clinical prognostic stratification for precision treatment as well as new research areas.

## Supplementary Information

**Additional file 1:** **Supplementary Table 1.** Description of data:Features identified from the feature selection step 2 and volume-derived metabolic parameters from step 1. **Supplementary Table 2.** Description of data: The average C-index with confidence interval of different modalities combinations and machine learning methods in the 1000 times bootstrap resampling training folds **Supplementary Table 3.** Description of data: The average C-index with confidence interval of different modalities combinations and machine learning methods in the 1000 times bootstrap resampling validation folds. **Supplementary Table 4.** Description of data: The range of hyperparameter tuning and R packages involved in this study. **Supplementary Figure 1.** Description of data: Radiomics feature selection step 2 with the least absolute shrinkage and selection operator (LASSO) cox regression model. (B, D) Tuning parameter selection in the LASSO model used five-fold cross-validation with minimum criteria for CT and PET respectively. Left vertical lines indicate the optimal value of the LASSO tuning parameter (λ). (A, C) LASSO coefficient profile plot with different log (λ). Vertical dashed lines represent radiomics features with nonzero coefficients screened with the optimal λ value for CT and PET respectively. **Supplementary Figure 2.** Description of data: The Kaplan-Meier analyses of all the MM patients based on the clinical hazard stratifications. Here, (A), (B) and (C) presented the cytogenetic abnormality, Riss_staging, Iss-staging, respectively.

## Data Availability

Since the raw and processed imaging data are also used in an ongoing study, it is not currently possible to share them in order to replicate these results. Contacting the respective author(Yue Chen) should be done in order to request access to these datasets.

## Abbreviations

^18^F-FDG PET: Quantitative 18F-fluorodeoxyglucose positron emission tomography
AUC: Area under the receiver operating characteristic curve
CT: Computed tomography
LASSO: Least Absolute Shrinkage and Selection Operator
FBW: Fixed bin width
VOIs: Volumes of interest
DCA: Decision curve analysis
AIC: Akaike information criterion
VIF: Variance inflation factor
TLG: Total Lesion Glycolysis
sTLG: Standardized Total Lesion Glycolysis = TLG/PatientWeight
MTV: Metabolic Tumor Volume
sMTV: Standardized Metabolic Tumor Volume = MTV/PatientWeight
ML: Machine learning
C-index: Harrell concordance index
GFR: Glomerular filtration rate
