# Supplementary material for: 18F‑FDG PET/CT based radiomics features improve prediction of prognosis: multiple machine learning algorithms and multimodality applications for multiple myeloma
Source: BMC Med Imaging. 2023 Jun 27;23:87. doi: 10.1186/s12880-023-01033-2 (PMC10303834; doi:10.1186/s12880-023-01033-2)
Supplement: Supplementary file 1 — Additional file 1: Supplementary Table 1. Description of data:Features identified from the feature selection step 2 and volume-derived metabolic parameters from step 1. Supplementary Table 2. Description of data: The average C-index with confidence interval of different modalities combinations and machine learning methods in the 1000 times bootstrap resampling training folds Supplementary Table 3. Description of data: The average C-index with confidence interval of different modalities combinations and machine learning methods in the 1000 times bootstrap resampling validation folds. Supplementary Table 4. Description of data: The range of hyperparameter tuning and R packages involved in this study. Supplementary Figure 1. Description of data: Radiomics feature selection step 2 with the least absolute shrinkage and selection operator (LASSO) cox regression model. (B, D) Tuning parameter selection in the LASSO model used five-fold cross-validation with minimum criteria for CT and PET respectively. Left vertical lines indicate the optimal value of the LASSO tuning parameter (λ). (A, C) LASSO coefficient profile plot with different log (λ). Vertical dashed lines represent radiomics features with nonzero coefficients screened with the optimal λ value for CT and PET respectively. Supplementary Figure 2. Description of data: The Kaplan-Meier analyses of all the MM patients based on the clinical hazard stratifications. Here, (A), (B) and (C) presented the cytogenetic abnormality, Riss_staging, Iss-staging, respectively. [file 12880_2023_1033_MOESM1_ESM.docx]

**Supplementary Materials**

**1. Supplementary Table 1**

**Title of data: Supplementary Table 1**

**Description of data:Features identified from the feature selection step 2 and volume-derived metabolic parameters from step 1.**

**File name: Supplementary Table 1.docx**

| Features | HR | z | pvalue | lower | upper |
| --- | --- | --- | --- | --- | --- |
| Globulins | 1.476 | 2.305 | 0.021 | 1.060 | 2.054 |
| A/G ratio | 0.543 | -2.588 | 0.010 | 0.342 | 0.862 |
| Red blood cells | 0.613 | -2.329 | 0.020 | 0.406 | 0.925 |
| HCT | 0.669 | -1.967 | 0.049 | 0.448 | 0.999 |
| Albumin | 0.447 | -3.911 | 0.000 | 0.299 | 0.669 |
| Hemoglobin | 0.627 | -2.204 | 0.028 | 0.415 | 0.950 |
| B2M | 1.425 | 2.825 | 0.005 | 1.115 | 1.823 |
| Absolute Neutrophils | 0.597 | -2.129 | 0.033 | 0.371 | 0.960 |
| ISS_staging | 2.581 | 3.437 | 0.001 | 1.503 | 4.433 |
| RISS_staging | 2.482 | 3.908 | 0.000 | 1.573 | 3.917 |
| Adverse prognostic cytogenetics status | 2.197 | 3.747 | 0.000 | 1.455 | 3.315 |
| wavelet.LLL_firstorder_10Percentile_PET | 1.796 | 2.881 | 0.004 | 1.206 | 2.674 |
| exponential_glcm_InverseVariance_PET | 1.996 | 3.396 | 0.001 | 1.339 | 2.973 |
| gradient_glszm_LargeAreaLowGrayLevelEmphasis_PET | 1.486 | 3.212 | 0.001 | 1.167 | 1.891 |
| lbp.2D_firstorder_InterquartileRange_PET | 1.470 | 2.765 | 0.006 | 1.119 | 1.932 |
| lbp.3D.m1_firstorder_RootMeanSquared_PET | 1.595 | 3.125 | 0.002 | 1.190 | 2.139 |
| lbp.3D.k_glcm_MCC_PET | 1.479 | 2.159 | 0.031 | 1.037 | 2.110 |
| original_shape_LeastAxisLength_PET | 1.777 | 2.872 | 0.004 | 1.200 | 2.630 |
| original_shape_MajorAxisLength_PET | 1.562 | 2.231 | 0.026 | 1.056 | 2.312 |
| original_shape_MinorAxisLength_PET | 1.653 | 2.904 | 0.004 | 1.177 | 2.320 |
| original_shape_MinorAxisLength_CT | 1.704 | 3.045 | 0.002 | 1.209 | 2.401 |
| original_ngtdm_Busyness_CT | 1.378 | 2.789 | 0.005 | 1.100 | 1.726 |
| exponential_glszm_SmallAreaLowGrayLevelEmphasis_CT | 0.492 | -2.997 | 0.003 | 0.309 | 0.782 |
| gradient_glcm_MCC_CT | 1.550 | 2.815 | 0.005 | 1.142 | 2.103 |
| squareroot_firstorder_90Percentile_CT | 1.539 | 3.705 | 0.000 | 1.225 | 1.933 |
| wavelet.HLH_gldm_SmallDependenceEmphasis_CT | 0.545 | -2.442 | 0.015 | 0.335 | 0.887 |
| wavelet.HHH_gldm_SmallDependenceEmphasis_CT | 0.552 | -2.449 | 0.014 | 0.343 | 0.888 |
| sMTV/ml.Kg^*^ | 1.408 | 2.208 | 0.027 | 1.039 | 1.908 |
| MTV/mL^*^ | 1.479 | 2.554 | 0.011 | 1.095 | 1.998 |
| MTV/voxel. ^*^ | 1.358 | 2.172 | 0.030 | 1.030 | 1.791 |
| sTLG/SUV.mLKg^*^ | 1.012 |  | 0.083 | 0.998 | 1.025 |
| TLG/SUV.mL^*^ | 1.000 |  | 0.073 | 1.000 | 1.000 |

* volume-derived metabolic parameters were screened from step 1

**2. Supplementary Table 2**

**Title of data: Supplementary Table 2**

**Description of data: The average C-index with confidence interval of different modalities combinations and machine learning methods in the 1000 times bootstrap resampling training folds**

**File name: Supplementary Table 2.docx**

| PET | PET_CLI | CT | CT_CLI | CLI |  |
| --- | --- | --- | --- | --- | --- |
| 0.837(0.835-0.840) | 0.917(0.916-0.919) | 0.793(0.791-0.796) | 0.910(0.908-0.912) | 0.838(0.836-0.840) | COX |
| 0.951(0.950-0.952) | 0.961(0.960-0.962) | 0.941(0.941-0.942) | 0.957(0.956-0.958) | 0.932(0.931-0.933) | RSF |
| 0.949(0.948-0.950) | 0.949(0.948-0.950) | 0.917(0.916-0.919) | 0.961(0.961-0.962) | 0.924(0.923-0.925) | GBM |
| 0.846(0.844-0.847) | 0.868(0.866-0.870) | 0.801(0.799-0.804) | 0.867(0.865-0.869) | 0.844(0.842-0.846) | SVRC |
| 0.840(0.837-0.842) | 0.920(0.918-0.921) | 0.793(0.790-0.796) | 0.906(0.904-0.908) | 0.839(0.836-0.841) | CoxBoost |
| 0.840(0.837-0.842) | 0.907(0.905-0.909) | 0.793(0.790-0.796) | 0.894(0.892-0.896) | 0.836(0.834-0.838) | GB-Cox |

**3. Supplementary Table 3**

**Title of data: Supplementary Table 3**

**Description of data: The average C-index with confidence interval of different modalities combinations and machine learning methods in the 1000 times bootstrap resampling validation folds.**

**File name: Supplementary Table 3.docx**

| PET | PET_CLI | CT | CT_CLI | CLI |  |
| --- | --- | --- | --- | --- | --- |
| 0.815(0.814-0.816) | 0.866(0.865-0.867) | 0.771(0.770-0.772) | 0.857(0.856-0.858) | 0.798(0.797-0.780) | COX |
| 0.858(0.857-0.860) | 0.880(0.878-0.881) | 0.837(0.836-0.839) | 0.871(0.869-0.872) | 0.850(0.848-0.851) | RSF |
| 0.855(0.854-0.857) | 0.868(0.866-0.869) | 0.819(0.817-0.820) | 0.876(0.875-0.878) | 0.837(0.836-0.838) | GBM |
| 0.793(0.792-0.795) | 0.845(0.843-0.846) | 0.744(0.742-0.746) | 0.832(0.831-0.833) | 0.794(0.793-0.780) | SVRC |
| 0.815(0.814-0.815） | 0.867(0.866-0.868) | 0.773(0.772-0.774) | 0.858(0.857-0.859) | 0.780(0.798-0.801) | CoxBoost |
| 0.814(0.813-0.814) | 0.863(0.862-0.864) | 0.772(0.771-0.773) | 0.851(0.853-0.855) | 0.801(0.780-0.8020) | GB-Cox |

**4. Supplementary Table 4**

**Title of data: Supplementary Table 4**

**Description of data: The range of hyperparameter tuning and R packages involved in this study.**

**File name: Supplementary Table 4.docx**

| COX | - | - | survival |
| --- | --- | --- | --- |
| RSF | Number of trees  Tterminal node size of forest | [1-1000]  [1-15] | randomForestSRC |
| GBM | Number of trees | [1-1000] | gbm |
| SVRC | Parameters of regularization  Kernel | [0.01-2]  “lin_kernel,add_kernel,rbf_kernel,ploy_kernel” | survivalsvm |
| CoxBoost | Number of boosting steps | [1-1000] | CoxBoost |
| GB-Cox | Number of boosting steps | [1-1000] | mboost |

**5.The detailed treatment combinations and cytogenetic abnormalities.**

In our study, based line treatment arms were developed depending on the patient's own primary diseases status and tolerance for treatments and regimens that recommended by the International Myeloma Working Group (IMWG) guidelines including[1]:

BCD: Bortezomib+ Cyclophosphamide + Dexamethasone 31;

BD: Bortezomib + Dexamethasone 7;

BDT: Bortezomib + Dexamethasone + Thalidomide 7;

MPT: Melphalan + Thalidomide + Prednisone 3;

PAD: Bortezomib + Doxorubicin + Dexamethasone 28;

PDT: Bortezomib + Pirarubicin + Dexamethasone 4;

PDD: Liposome doxorubicin + Bortezomib + Dexamethasone 4;

TAD: Dexamethasone + Thalidomide + Epirubicin 3;

TD: Dexamethasone + Thalidomide 1;

VMP: Bortezomib + Melphalan + Dexamethasone 1;

VRD: Bortezomib + Lenalidomide + Dexamethasone 9;

Cytogenetics analysis Fluorescence in situ hybridization (FISH) was performed in 98 patients, adverse prognosis cytogenetics status was defined as gain 1q21, t(4;14), del 17p, t(14;16) according to Dimitrios C. Ziogas et alv and Pieter Sonneveld et al. [2-3] Total 79 cytogenetics abnormities in 41 patients were detected including gain 1q21(n=32), del 17p (n=12), t(4;14) (n=9), del 1p32 (n=3), t(14;16) (n=4), t(14;20) (n=4), del (13q14) (n=12), t(11;14) (n=3).

**6. Supplementary Figure 1**

**Title of data: Supplementary Figure 1**

**Description of data: Radiomics feature selection step 2 with the least absolute shrinkage and selection operator (LASSO) cox regression model. (B, D) Tuning parameter selection in the LASSO model used five-fold cross-validation with minimum criteria for CT and PET respectively. Left vertical lines indicate the optimal value of the LASSO tuning parameter (λ). (A, C) LASSO coefficient profile plot with different log (λ). Vertical dashed lines represent radiomics features with nonzero coefficients screened with the optimal λ value for CT and PET respectively.**

**File name: LASSO.tif**


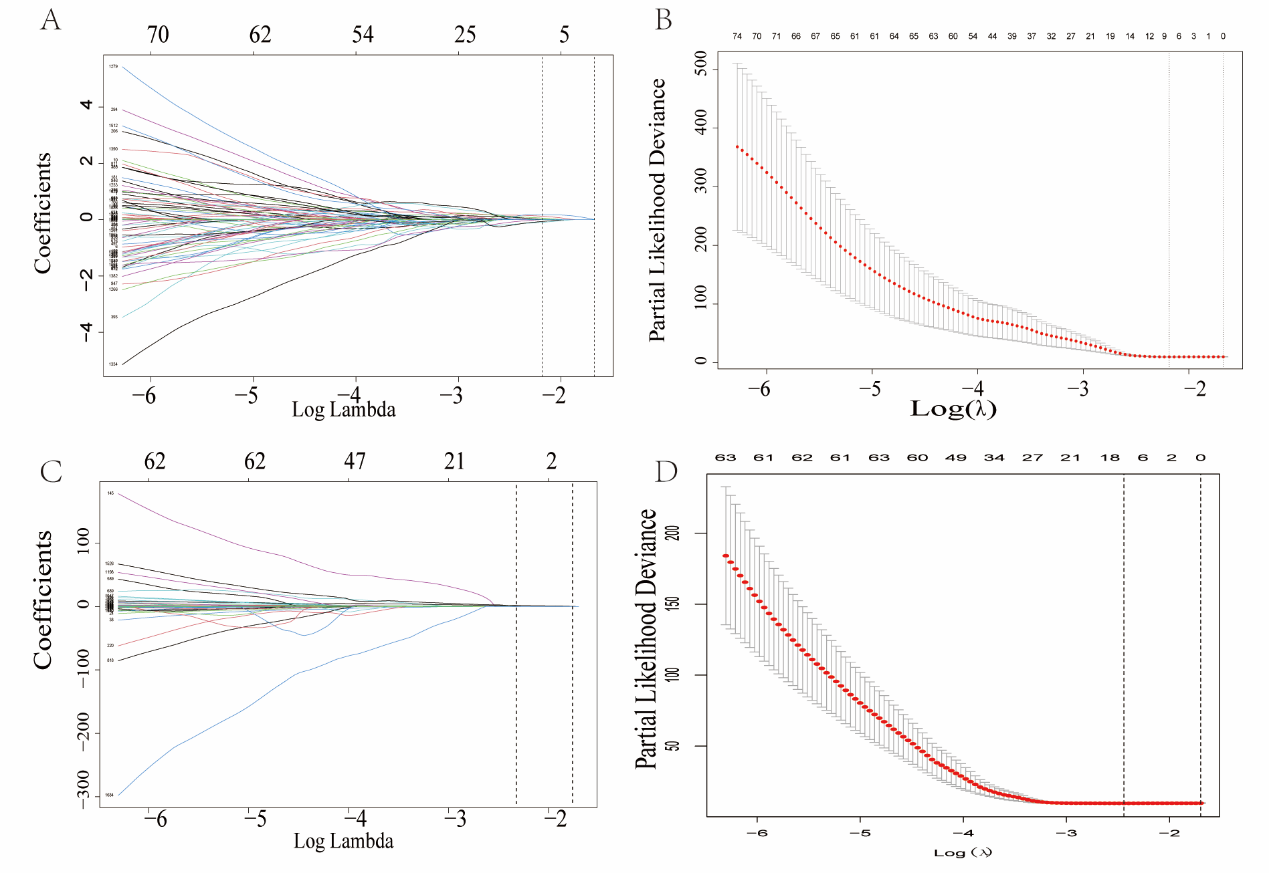


**7. Supplementary Figure2**

**Title of data: Supplementary Figure2**

**Description of data: The Kaplan-Meier analyses of all the MM patients based on the clinical hazard stratifications. Here, (A), (B) and (C) presented the cytogenetic abnormality, Riss_staging, Iss-staging，respectively**

**File name: Survival.tif**


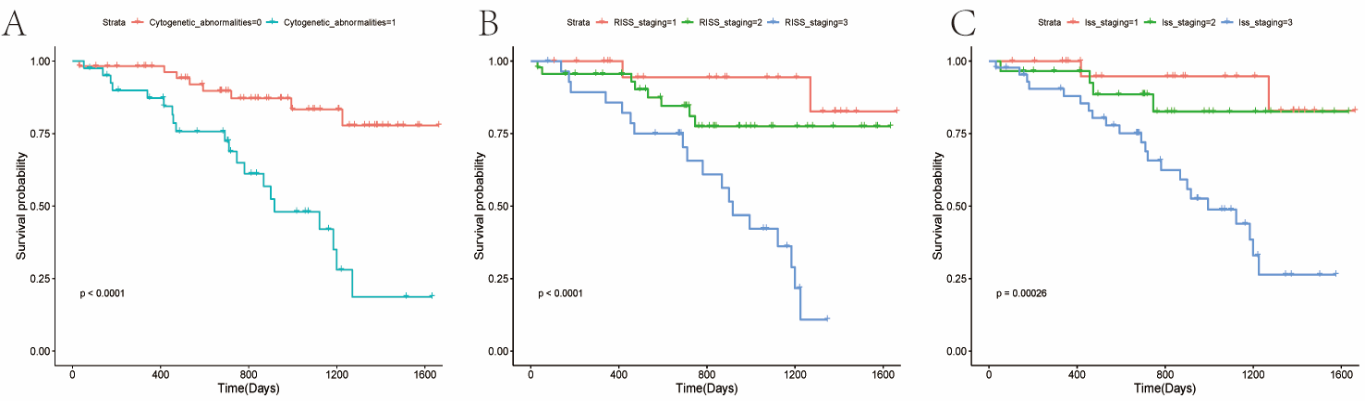


8.

**The definitions of progression and relapse are based on the International Myeloma Working Group consensus criteria for response and minimal residual disease assessment in multiple myeloma.**

Clinical relapse requires one or more of the following criteria:Development of new soft tissue plasmacytomas or bone lesions (osteoporotic fractures do not constitute progression); Definite increase in the size of existing plasmacytomas or bone lesions. A defi nite increase is defi ned as a 50% (and ≥1 cm) increase as measured serially of the measurable lesion; Hypercalcaemia (>11 mg/dL); Decrease in haemoglobin of ≥2 g/dL not related to therapy or other non-myeloma-related conditions; Rise in serum creatinine by 2 mg/dL or more from the start of the therapy and attributable to myeloma; Hyperviscosity related to serum paraprotein.

Progressive disease:Any one or more of the following criteria: Increase of 25% from lowest confi rmed response value in one or more of the following criteria: Serum M-protein (absolute increase must be ≥0·5 g/dL); Serum M-protein increase ≥1 g/dL, if the lowest M component was ≥5 g/dL; Urine M-protein (absolute increase must be ≥200 mg/24 h); In patients without measurable serum and urine M-protein levels, the diff erence between involved and uninvolved FLC levels (absolute increase must be >10 mg/dL); In patients without measurable serum and urine M-protein levels and without measurable involved FLC levels, bone marrow plasma-cell percentage irrespective of baseline status (absolute increase must be ≥10%); Appearance of a new lesion(s), ≥50% increase from nadir in SPD§§ of >1 lesion, or ≥50% increase in the longest diameter of a previous lesion >1 cm in short axis; ≥50% increase in circulating plasma cells (minimum of 200 cells per μL) if this is the only measure of disease.[4]

**Reference**

1.Kumar SK, Mikhael JR, Buadi FK, et al. **Management of newly diagnosed symptomatic multiple myeloma: Updated Mayo stratification of myeloma and risk-adapted therapy (mSMART) consensus guidelines**. Mayo Clin Proc 2009;84(12):1095-1110

2.Ziogas DC, Dimopoulos MA, Kastritis E. **Prognostic factors for multiple myeloma in the era of novel therapies**. DOI:10.1080/17474086.2018.1537776.

3.Pieter Sonneveld, Hervé Avet-Loiseau, Sagar Lonial, Saad Usmani,David Siegel et al. **Treatment of multiple myeloma with high-risk cytogenetics: a consensus of the International Myeloma Working Group**. DOI:10.1182/blood-2016-01-631200.

4.Kumar S, Paiva B, Anderson KC, et al. **International Myeloma Working Group consensus criteria for response and minimal residual disease assessment in multiple myeloma.** Lancet Oncol. 2016;17(8):e328-e346. doi:10.1016/S1470-2045(16)30206-6
